# Neuronal Differentiation in a Pituitary Macroadenoma with Focal Small Blue Round Cell Morphology: Report of a Rare Pattern

**DOI:** 10.1155/2020/6450930

**Published:** 2020-04-29

**Authors:** Hisham Alkhalidi

**Affiliations:** Department of Pathology, College of Medicine, King Saud University, Riyadh, Saudi Arabia

## Abstract

Ganglion cell-containing pituitary adenomas that are neurofilament protein-positive are the exceedingly rare form of pituitary ganliocytomas. We report a case of a 23-year-old male patient who presented with a clinical picture of acromegaly in addition to raised prolactin level. Histopathology showed areas exhibiting pseudopapillary and solid proliferation of round and monotonous blue cells. The immunohistochemistry showed strong reactivity for synaptophysin and growth hormone and focal reactivity to prolactin. Fibrous bodies are confirmed using cytokeratin immunostain, in keeping with sparsely granulated somatotroph component. The patient remained free of recurrence after one year of radiological follow-up.

## 1. Introduction

Pituitary macroadenomas are benign pituitary gland tumors with an estimated 10-25% overall prevalence among all intracranial neoplasms that grow to at least 1 cm in size [1, 2]. They are classified based on their size, immunohistologic characterization, and functional criteria (i.e., serum hormone levels) [3]. Pituitary adenomas usually secrete prolactin, growth hormone (GH), and other hormones including adrenocorticotropic hormone in both men and women [4]. In this report, we present a very rare case of GH-secreting mixed gangliocytoma-pituitary adenoma that exhibits rare and dominant neuronal differentiation associated with atypical cellular morphology and focal reactivity to prolactin.

## 2. Case Report

**Figure 1:**
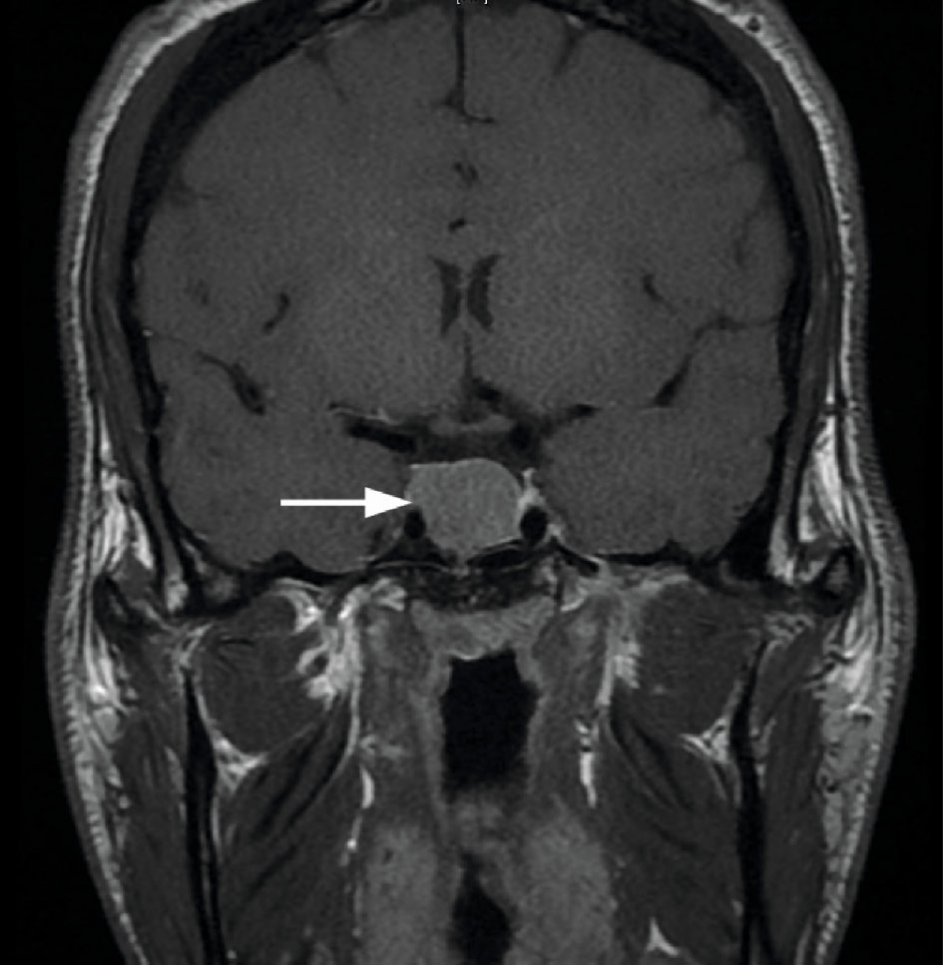
Magnetic resonance imaging (MRI). The sella was expanded by a macroadenoma (white arrow) with mild contrast enhancement.

A 23-year-old male patient presented with a clinical picture of acromegaly. GH level was 40.33 ng/mL (reference < 10 ng/mL), insulin-like growth factor 1 (IGF-1) was 1056 ng/mL (reference 96-228 ng/mL), and prolactin was 759 mIU/L (reference 86-324 mIU/L). Magnetic resonance imaging showed that the sella turcica was expanded by a pituitary macroadenoma measuring 2.1 × 1.8 × 1.9 cm (Figure 1). Mild enhancement after contrast material injection was noted. There was no extension to the suprasellar cistern, cavernous sinuses, or sphenoid sinus. However, the patient showed calvarial thickening and frontal bossing, in keeping with the diagnosis of acromegaly.

**Figure 2:**
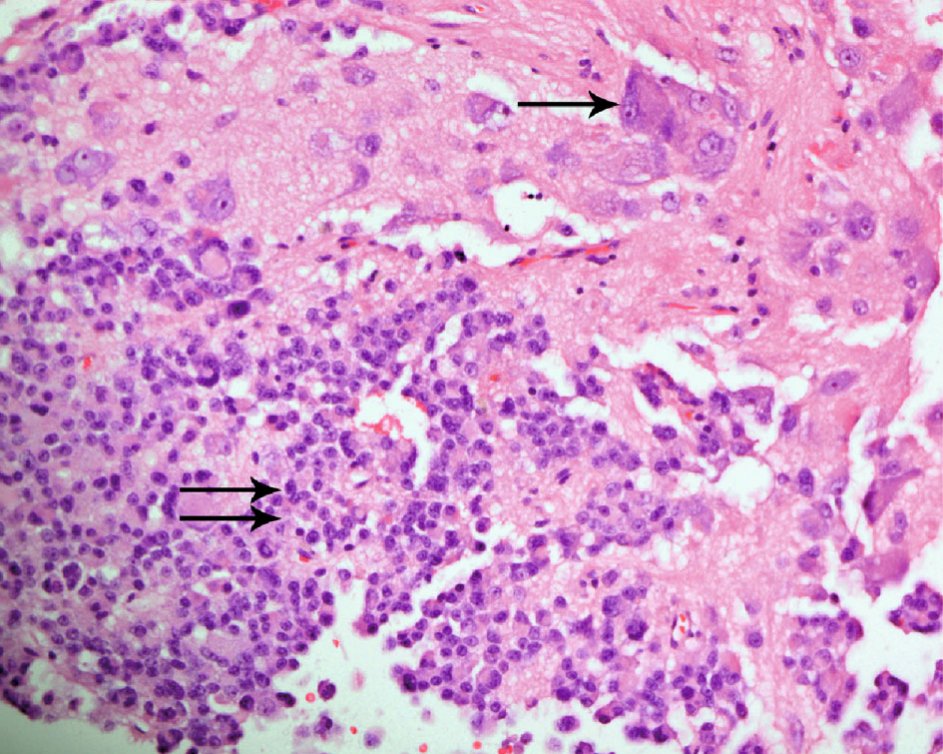
The tumor has two areas. One dominated by round monotonous cells (double arrows) forming pseudopapillary structures and another (upper area) showing neuropil tissue where scattered neurons (single arrow) are noted (H&E stain, X100).

**Figure 3:**
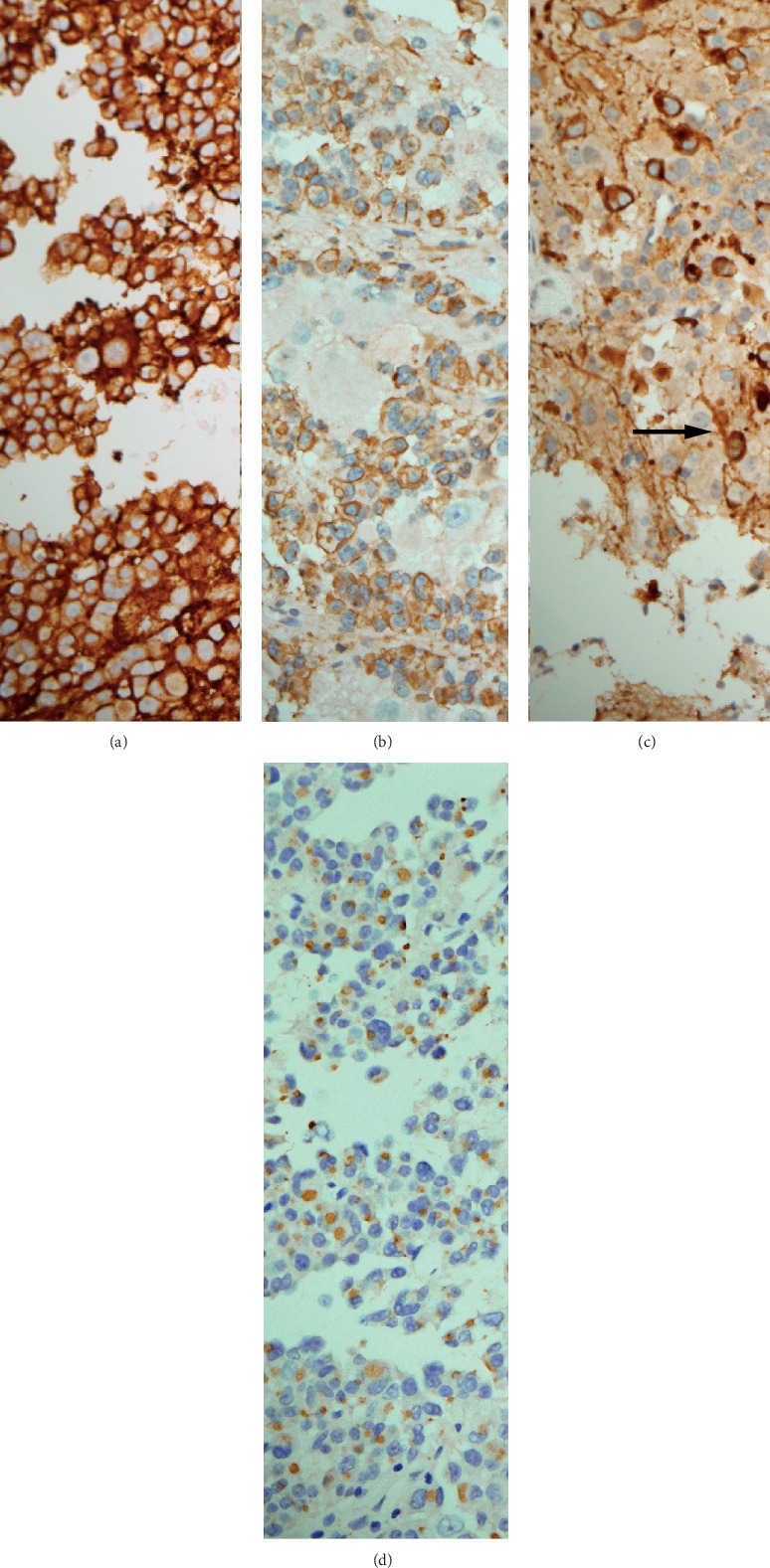
(a) Synaptophysin immunohistochemical stain highlights the cytoplasm of the tumor cells (×200). (b) Growth hormone immunohistochemical stain highlighting most of the tumor cells (×200). (c) Small neurons and their axons (arrow) showing strong reactivity to prolactin immunohistochemical stain (×200). (d) Cytokeratin paranuclear dot-like staining depicts fibrous bodies in most of the tumor cells cytoplasm (×200).

The tumor was resected using a transnasal approach. Histopathological examination of the resected tumor samples showed two patterns (Figure 2). One was an area that was neuropil-rich, while the second area showed small monotonous cells exhibiting pseudopapillary and solid proliferation. In the latter area, the nuclei of the tumor cells were round with little cytoplasm. This proliferation interfaced with the neuropil-rich tissue that had conspicuous scattered neurons. No mitotic activity or necrosis was detected. Immunohistochemical studies (Figure 3) showed that the tumor cells are strongly positive for synaptophysin and growth hormone. Focal reactivity to prolactin or chromogranin was present. The prolactin was staining the neuron cell bodies and their attached cell processes. FSH, LH, ACTH, and TSH were negative. Cytokeratin revealed paranuclear inclusions (fibrous bodies). GFAP was negative in the tumor cells. INI-1 exhibited normal nuclear reactivity pattern. Ki-67 proliferative index was low. A 1-year follow-up of the patient using MRI revealed no recurrence of the tumor.

## 3. Discussion

Ganglion cell-containing pituitary adenomas are very rare and usually show neurofilament protein- (NFP-) positive, dot-like areas of cytoplasmic reactivity that indicates neuronal differentiation within adenoma cells [5–7]. These tumors usually regress spontaneously in approximately 10% of patients, and observation alone saves the patient a surgical intervention [8]. However, the progression of the tumor is observed in 50% of patients in whom the tumor was not resected. Recurrence of the tumor is reported in 6–46% of patients after transsphenoidal surgery [9, 10].

Histopathology of the resected tumor in our patient showed round blue cell areas that look atypical on low power. These areas are located at the edges of the neuropil-rich area. Round cells are typically noted in sparsely granulated somatotroph pituitary adenoma. The tumor cells in this case also showed paranuclear inclusion (fibrous bodies) on Cytokeratin stain, which is a feature of sparsely granulated somatotroph. No mitosis or necrosis was seen, and the Ki-67 proliferative index was low. This may indicate a lower probability of postsurgical progression. Some studies have shown that immunohistochemistry for Ki-67 of pituitary adenomas with a proliferation index greater than 1.3% predicts the progression of the disease [11]. However, according to the new WHO classification of 2017, Ki-67 is no longer a reliable prognostic factor.

An interesting feature in this case is the prolactin reactivity, which was highlighting neurons and their sprouting axons. Hence, the increased serum prolactin level was probably due to the prolactin secretion of the neuronal compartment of the tumor.

As per the WHO classification, this is a case of mixed gangliocytoma-pituitary adenoma. Different theories for the origin of pituitary adenomas with neuronal elements are suggested. Thodou et al. [12] summarized these theories into three categories. The first one accepts the existence of neuronal elements, possibly as embryonic remnants in the adenohypophysis and the development of the two lesions being incidental with no relation. These tumors may originate in remnants, showing intermediate features of neuronal-adenohypophysial cells. The second theory is that heterotopic hypothalamic neurons within the adenohypophysis can produce stimulatory peptides (for example growth hormone releasing hormone) leading to hyperplasia and subsequent neoplastic transformation of adenohypophysial cells. A recent publication by Teramoto et al. [13] revealed colocalization of GH and GH-releasing hormone (GHRH) in pituitary adenoma cells. The third hypothesis suggests the pituitary adenoma as the initial lesion. Some adenoma cells under unknown stimulatory mechanisms can be transformed into neuronal elements. A DNA analysis and chromosomal study have demonstrated the monoclonal origin of these tumors and concluded that these tumors arise from a genetic mutation in a single cell [14].

In our case, the tumor showed prolactin stain highlighting small cells that exhibit attenuated neuronal axons. In a case series by Lopes et al. [15], the authors demonstrated that both adenomatous and ganglionic cells could express at least a small population of cells that coexpress the Pit-1 transcription factor and neuronal-associated cytoskeletal proteins. This favors the theory of transdifferentiation of neuroendocrine cells into neuronal elements of these mixed tumors.

Long-term monitoring of these patients may be necessary although most pituitary adenomas are generally slow-growing with low proliferative index. It was suggested that if no evidence of tumor progression is shown after 3-5 years following surgery, monitoring of the patient should be extended for an additional period [10].
